# ChMER: an exoskeleton robot with active body weight support walker based on compliant actuation for children with cerebral palsy

**DOI:** 10.3389/fbioe.2025.1551039

**Published:** 2025-02-27

**Authors:** Yuantao Ding, Zhengtao Wang, Peizhong Yang, Suiran Yu

**Affiliations:** The State Key Laboratory of Mechanical System and Vibration, School of Mechanical Engineering, Shanghai Jiao Tong University, Shanghai, China

**Keywords:** rehabilitation robotics, pediatric exoskeleton, active body weight support system, compliant actuation, cerebral palsy

## Abstract

**Introduction:**

Lower limb exoskeleton robots for young children with cerebral palsy (CP) are crucial to support earlier rehabilitation that is more beneficial than later. For safety reasons, pediatric exoskeletons are usually equipped with body weight support (BWS) devices to help young patients maintain balance. However, existing pediatric exoskeletons tend to use stiff joint actuation and passive BWS with limited compliance.

**Method:**

This paper proposes a novel mobile exoskeleton robot for young children (3- ∼ 6-years-old) with CP based on intrinsically compliant actuation. A compact kinematic chain that integrates an exoskeleton, an active BWS system, and a walker is proposed. Furthermore, with the actuation design optimization of the kinematic chain, the robot can walk alone stably in passive rehabilitation and provide high compliance in active rehabilitation. The exoskeleton adopts actuation similar to the quasi-direct drive paradigm to acquire high mechanical compliance and uses a secondary planetary reducer to ensure high output torque. Assistive torque control is achieved through proprioceptive sensing instead of torque sensors. The BWS system uses a series elastic actuator to accurately generate the weight support force and significantly reduce the fluctuation of the support force compared to the passive BWS.

**Results and discussion:**

Finally, control frameworks for passive and active rehabilitation are implemented to validate the robot performance. The experimental results demonstrate that our robot can support safe and compliant rehabilitation.

## 1 Introduction

Cerebral palsy (CP) is a common movement disorder in children, affecting approximately 1.6% ∼ 3.4% of newborns (McIntyre et al., 2022), severely impairing their ordinary life and growth. Conventional treatment depends on the experience and labor of rehabilitation therapists (Aisen et al., 2011). However, with the development of rehabilitation robotics, new approaches have emerged, opening up new possibilities for enhancing pediatric rehabilitation. For instance, several clinical studies have demonstrated the effectiveness of robot-assisted gait therapy (RAGT) (Jin et al., 2020) and partial body weight support treadmill training (PBWSTT) (Willoughby et al., 2010) in improving postural and motor function in children with motor impairments. In recent years, some lower limb orthoses and exoskeletons have been developed for children with CP, offering the potential to supplement traditional physical rehabilitation (Gonzalez et al., 2021; Sarajchi et al., 2021).

Stationary gait rehabilitation systems, such as Lokomat (Wallard et al., 2017) and Walkbot (Jin et al., 2020), were the first to be clinically applied. They assist patients with natural gait rehabilitation through the coordinated movement of the exoskeleton and the treadmill. However, their high cost and large size limit them to rehabilitation clinics. Wearable exoskeletons (Patané et al., 2017; Lerner et al., 2018; Eguren et al., 2019) can provide gait correction while allowing patients to walk on the ground. They have the potential to facilitate home-based rehabilitation and offer greater benefits to pediatric patients (Ding et al., 2024a). Rehabilitation safety is often cited as a primary consideration (Wang et al., 2023). To ensure this safety, most wearable exoskeletons must be used with crutches for dynamic balance and fall prevention (Qiu et al., 2023). However, this is impractical for children with CP at a low age, who benefit more from earlier rather than later intervention (Patel et al., 2020). In order to maintain the balance of the human-robot system and facilitate earlier rehabilitation when patients are weak (Bayón et al., 2017), some studies have attached exoskeletons to support devices (Maggu et al., 2018; Llorente-Vidrio et al., 2020; Narayan and Kumar Dwivedy, 2021; Cumplido-Trasmonte et al., 2022). For example, ATLAS 2030 (Cumplido-Trasmonte et al., 2022) mounted the exoskeleton to a particular frame, while Trexo (Maggu et al., 2018) added the exoskeleton to a commercial walker. However, combining the exoskeleton with a simple support mechanism only provides passive body weight support (BWS) and limits flexibility at the attachment point.

Compliant human-robot interaction (HRI) is also essential for safety and comfort in rehabilitation (Gong et al., 2024). Compared to passive BWS, active BWS systems can compliantly control the body weight support force. However, they also increase the complexity of structure and control. Moreover, existing stationary BWS systems are usually complicated and heavy (Dong et al., 2021b; Mokhtarian et al., 2023), while mobile BWS systems (Dong et al., 2021a; Kwak et al., 2022; Stramel et al., 2023) are commonly developed separately without integrated exoskeletons. Therefore, it is still a challenge to integrate an exoskeleton with an active BWS system while remaining compact and lightweight. Most existing studies on the compliant control of pediatric exoskeletons have used low - torque motors with high gear ratios. These are also known as traditional stiff actuators (TSAs) (Bayón et al., 2017; Andrade et al., 2019). They are typically equipped with torque sensors to achieve active compliance through feedback control (Zhu et al., 2022), such as dynamic compensation control (Andrade et al., 2019) and impedance control (Bayón et al., 2017). However, TSAs are not backdrivable and lack mechanical compliance, motivating researchers to enhance the intrinsic compliance of the actuators from the mechanical design. For example, WAKE-Up (Patané et al., 2017) employed a series elastic actuator (SEA) to design the exoskeleton joint, which increased compliance by adding torsion springs and belts between the motor and the load.

Research on compliant actuation has mainly focused on adult rehabilitation so far. In this area, several novel actuation methods have been proposed to improve robotic performance. Variable stiffness actuators (VSAs) have been developed for both exoskeletons (Liu et al., 2024) and BWS systems (Dong et al., 2021a) to adjust the stiffness of the elastic element to match the stiffness requirements of different gait events. However, they inevitably increase the volume and mass of the robot, which poses a challenge in the confined space arrangement of pediatric rehabilitation robots. Quasi-direct drive (QDD) actuation (Ding et al., 2024b) uses high-torque motors and small transmission ratios (≤10:1), resulting in low mechanical impedance and high compliance. However, due to the small transmission ratio, the output torque is low and currently suitable for applications where only partial assistance is required. Although the design constraints of rehabilitation robots differ significantly between adults and children, these novel actuation paradigms also inspire the design of the pediatric exoskeleton and the active BWS system in this work.

**FIGURE 1:**
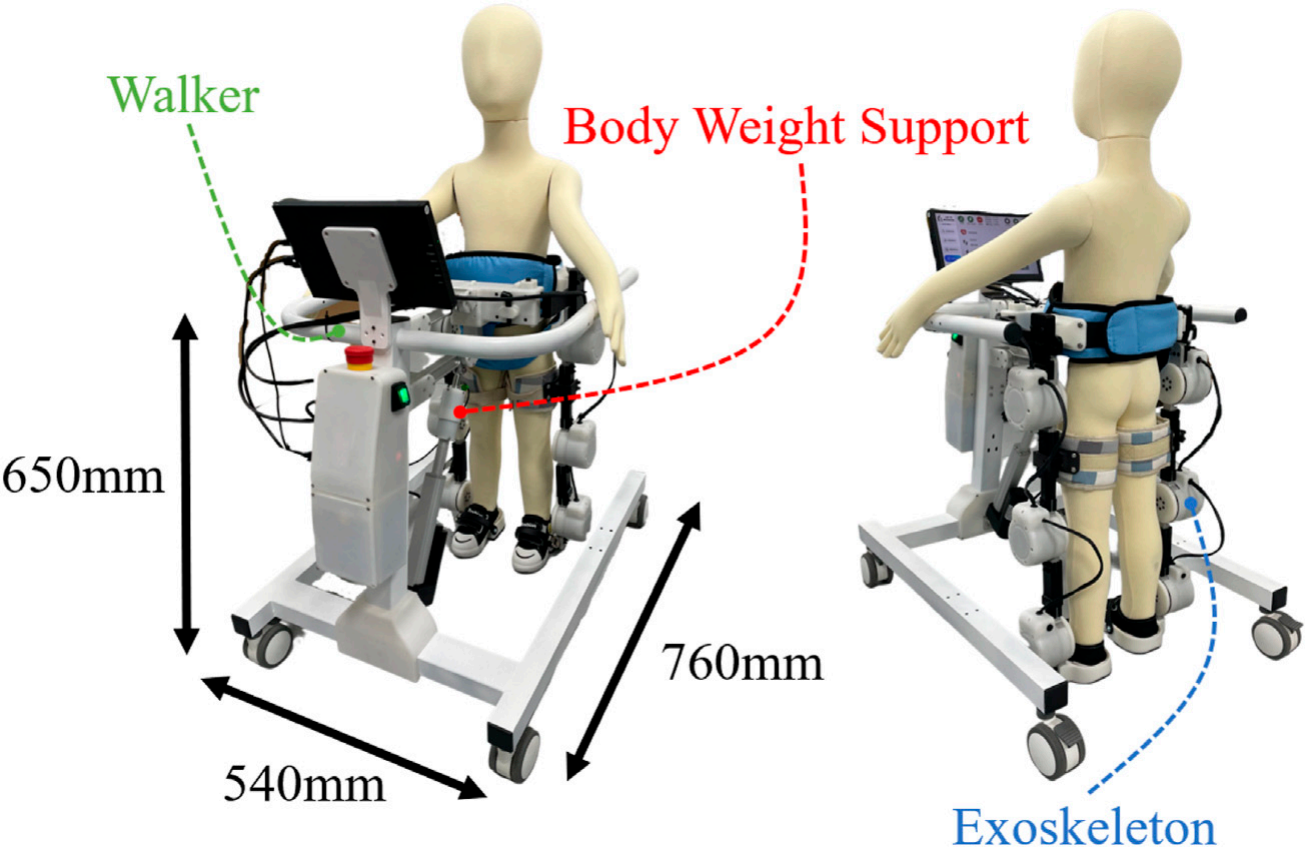
Overview of the proposed mobile exoskeleton robot (ChMER) with an active body weight support walker for young children with CP.

This paper presents a novel children’s mobile exoskeleton robot (hereafter referred to as ChMER, see Figure 1) for overground gait rehabilitation of young children with CP. Table 1 shows the comparison of ChMER with other similar pediatric rehabilitation robots, indicating that the existing robots mainly use passive BWS with limited compliance, while the robot (Bayón et al., 2017) with an active BWS system is unsuitable for young children. Moreover, although SEAs, VSAs, and QDD actuators have demonstrated excellent performance and application value in adult rehabilitation devices, TSAs are still dominant in the existing pediatric rehabilitation robots. Therefore, based on the intrinsically compliant actuation, this work focuses on the compact mechatronic design of ChMER with an active BWS system, which can accommodate the limited size of young children’s limbs while providing compliant and safe HRI. The main contributions of this work are: 1) proposing a compact kinematic chain that integrates an exoskeleton, an active BWS system, and a walker and designing the actuation patterns for passive and active rehabilitation; 2) developing and validating the pediatric exoskeleton and the active BWS system with the compliance-oriented design concept based on multifactorial trade-off analysis of the intrinsically compliant actuation. The exoskeleton adopts actuation similar to the QDD paradigm to improve mechanical compliance and uses a secondary planetary transmission to provide high output torque. Torque control is achieved through current-based proprioceptive torque sensing rather than torque sensors. The BWS system uses an SEA with a mechatronic design to accurately generate and sense the weight support force in the vertical direction. Furthermore, control frameworks for passive and active rehabilitation are implemented based on the above.

**TABLE 1:** Comparisons with some other mobile exoskeleton rehabilitation robots for children.

Name	Suitable age	BWS type	Actuated joint	Exoskeleton actuator	Torque sensing type
CPWalker (Bayón et al., 2017)	11∼18	Active	Hip, Knee, Ankle	Motor+160:1 reducer	Strain gauges
Trexo (Maggu et al., 2018)	—	Passive	Hip, Knee	Motor	No
Atlas2030 (Cumplido-Trasmonte et al., 2022)	3∼14	Passive	Hip, Knee, Ankle	SEA	Elastic elements
Dusthon et al. (Llorente-Vidrio et al., 2020)	10∼18	Passive	Hip, Knee, Ankle	Linear actuator	No
Jyotindra et al. (Narayan and Kumar Dwivedy, 2021)	8∼12	Passive	Hip, Knee	Lead screw actuator	No
This work	3∼6	Active	Hip, Knee, Ankle	Motor+36:1 reducer	Proprioception

## 2 Requirements analysis and integrated design

### 2.1 Requirements of ChMER

Clinical studies (Alriksson-Schmidt et al., 2017; Burgess et al., 2022) have shown that children with CP are expected to reach 90% of their motor - developmental potential by the age of five and then reach a plateau. Hence, early intervention is critical to optimize the motor function of the affected children (Damiano, 2006). Therefore, the target population for ChMER is selected to be 3- to 6-years-old children at Gross Motor Function Classification System (GMFCS) levels II to V. GMFCS levels range from I to V, with the motor function of children gradually deteriorating as the level increases. The design criteria for the robot are as follows. First, ChMER should adapt to the tiny limbs of young children and have an active BWS system to ensure safety and comfort during rehabilitation training while maintaining a simple structure to increase reliability and reduce cost. In addition, ChMER should provide a variable assistance mode to adapt to the different motor abilities of the patient. Specifically, children at GMFCS levels II and III have a certain degree of independent walking ability. In contrast, those at GMFCS levels IV and V usually cannot walk independently. Therefore, the robot should provide complete assistance for passive rehabilitation in children with no active motor ability (GMFCS levels IV and V) and adjustable compliant assistance for active rehabilitation in children with partial motor ability (GMFCS levels II and III).

According to the study of the peak joint torque of healthy children during normal walking (Chester et al., 2006), for the children weighing 25 kg, the peak torques of the hip, knee, and ankle joints are about 22.5, 17.5, and 37.5 Nm, respectively. Therefore, the peak output torque of the exoskeleton joint is required to reach these joint torques. Moreover, according to the lower limb size of three-year-old children (Tilley and Associates, 2002), the maximum diameter of the exoskeleton joint is limited to 90 mm. The BWS system should be able to reduce 70% of the children’s body weight. Therefore, it is required to provide a maximum weight unloading of approximately 175 N. The design requirements and actual parameters are summarized in Table 2.

**TABLE 2:** Design parameters of ChMER.

Parameters	Value
Suitable age	3–6 years-old
Peak torque of children weighing 25 kg (Hip/Knee/Ankle)	22.5/17.5/37.5 Nm
Max output torque of actuator (Desired/Actual)	37.5 Nm/42 Nm
Limb length of children aged 3 (Thigh/shank/ankle)	195/193/51 mm
Diameter of the exoskeleton joint (Desired/Actual)	<90/84 mm
Weight of children aged 6	250 N
Max BWS force (Desired/Actual)	175/200 N

### 2.2 Integrated design of exoskeleton, BWS system, and walker

**FIGURE 2:**
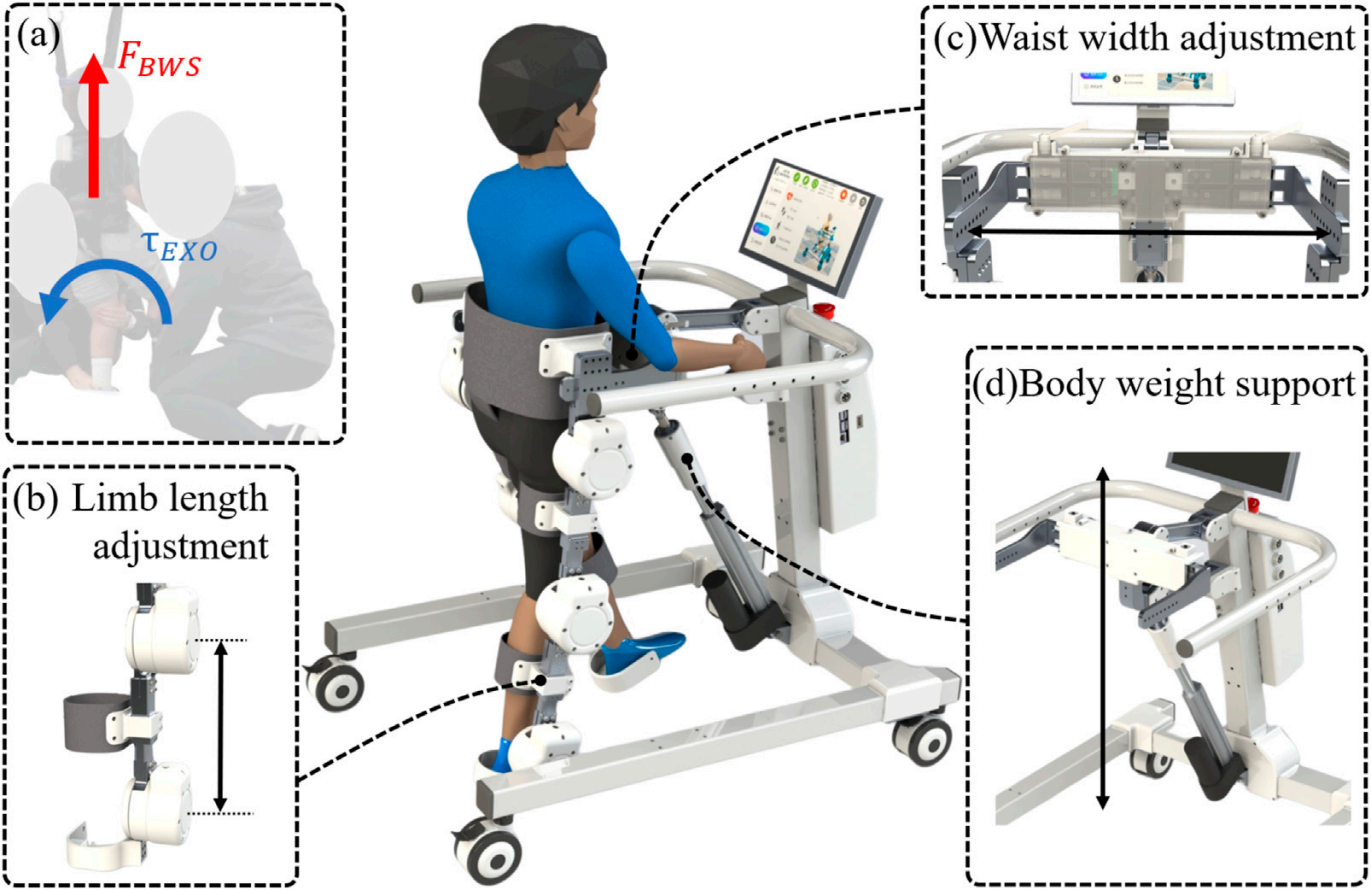
Basic structure of ChMER. **(A)** The design concept derived from the manual treatment. **(B)** The adjustment mechanisms of the limb length and **(C)** waist width. **(D)** The body weight support system.

The design concept of ChMER is derived from the manual treatment by a therapist, as illustrated in Figure 2A, which aims to provide joint assistive torque $\tau_{EXO}$ and vertical support force $F_{BWS}$ through the exoskeleton and the BWS system. The basic structure of ChMER is shown in Figure 2. The hip, knee, and ankle joints of the exoskeleton are actuated by motors (Figure 2B), while the BWS system is actuated by a linear actuator (Figure 2D). To fit the body size of different children, ChMER is equipped with the adjustment mechanisms of the limb length (Figure 2B) and waist width (Figure 2C). Specifically, the BWS system (Figure 2D) can both unload the children’s weight and adapt to the children’s height.

**FIGURE 3:**
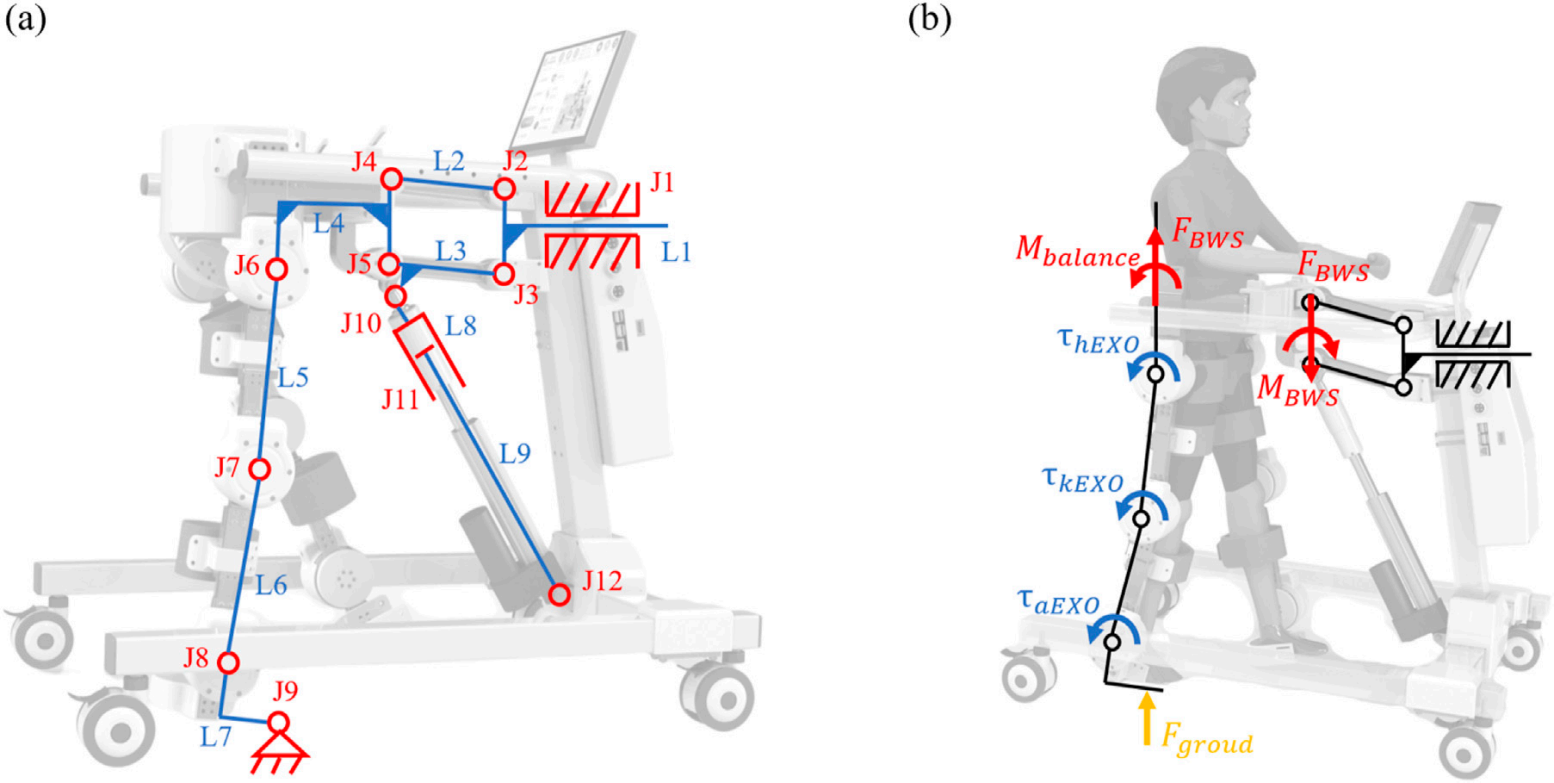
Kinematics and force diagram of ChMER. **(A)** Mechanism sketch of the compact kinematic chain. **(B)** Force diagram of the support leg during walking.

Unlike previous studies (Maggu et al., 2018) that design the exoskeleton and the BWS system separately, we integrate them into a continuous kinematic chain with a walker instead of isolated components, which enhances the system’s compactness. The mechanism sketch of the robot is shown in Figure 3A, where the walker is simplified as a translational joint ( $J_{1}$ ). The BWS system consists of a parallel four-bar structure ( $J_{2}∼J_{5}$ ) and a linear actuator ( $J_{10}∼J_{12}$ ). The exoskeleton comprises hip, knee, and ankle joints ( $J_{6}∼J_{8}$ ). The contact point between the support foot and the ground is simplified as a rotating joint ( $J_{9}$ ). The whole walker is simplified as a single rod $L_{1}$ . The rods of the parallel four-bar mechanism are denoted as $L_{1}∼L_{4}$ . $L_{5}∼L_{7}$ denote the thigh, calf and foot rods of the exoskeleton. $L_{8}∼L_{9}$ denote the two rods of the linear actuator. Therefore, the degrees of freedom (DoFs) of the system can be calculated as follows:

F=3×9−2×12=3 (1)

For passive rehabilitation, the robot needs to follow a predefined trajectory. In Equation 1, the 3 DoFs refer to the number of independent motion parameters that must be specified for the mechanism to have a definite motion. In other words, it is equal to the number of prime movers required to move the walker forward. Therefore, it is necessary to select three of the hip, knee, and ankle joints ( $J_{6}∼J_{8}$ ) and the linear actuator ( $J_{11}$ ) as the prime movers and the other as the passive DoF so that the system has deterministic motion. Some exoskeletons install springs in the ankle joint ( $J_{8}$ ) to act as the passive DoF. However, children with CP are prone to ankle abnormalities. The correction of which is also essential (Orekhov et al., 2020). Considering this, the three joints of the exoskeleton ( $J_{6}∼J_{8}$ ) are selected as the prime movers. Consequently, the linear actuator ( $J_{11}$ ) should work in force control instead of position control to avoid generating additional actuation. Thus, the system has no over-constraints or redundant DoFs. In this case, a simple striding motion of the exoskeleton can propel the walker so that the robot can walk stably on the ground by itself without the need for the patient to maintain the stability of the human-robot system, which effectively simplifies the control algorithms while ensuring rehabilitation safety. In addition, the active BWS system allows the four-bar mechanism to automatically adapt to the fluctuation of the system’s center of mass height (CoMH) during walking. In contrast, the support force $F_{BWS}$ of the passive BWS system fluctuates with the CoMH, which gives the patient a sense of undulation of weightlessness or overweight and limits the rehabilitation effect (Mirzaee et al., 2019).

For active rehabilitation, the movement of the system is dominated by the children, requiring ChMER to be able to apply assistive forces as needed. The diagram of the dynamic analysis of the human-robot system during walking is shown in Figure 3B. Considering the support leg as tandem type joints and the waist as the base, the coupled human-robot dynamic model of the support leg can be obtained as follows:

$$M\left(q\right)\ddot{q}+C\left(q,\dot{q}\right)+G\left(q\right)+F\left(\dot{q}\right)=\tau_{EXO}+\tau_{human}-J^{T}\left(q\right)F_{groud}$$ (2)

where $M\left(q\right)$ is the combined inertia matrix of the exoskeleton and the user, $C\left(q,\dot{q}\right)$ is the centripetal matrix, $G\left(q\right)$ is the gravity matrix, $F\left(\dot{q}\right)$ is the friction matrix, $\tau_{EXO}$ is the output torque of the exoskeleton joints, $\tau_{human}$ is the joint torque of the user, $J\left(q\right)$ is the Jacobi matrix of the robot, and $F_{groud}$ is the ground support reaction force acting on the end of the exoskeleton. Neglecting the acceleration of the system due to the slow motion of rehabilitation, $F_{groud}$ can be approximated as the total gravity of the human-robot system Mg minus $F_{BWS}$ :

$$F_{groud}=Mg-F_{BWS}$$ (3)

From Equations 2 and 3, it can be seen that $\tau_{EXO}$ directly applies assistive torque to the joints, and $F_{BWS}$ indirectly provides assistance by reducing the ground support reaction force. If the robot can precisely generate $\tau_{EXO}$ and $F_{BWS}$ , which can also be called completely transparent (Woo et al., 2017), it can exhibit arbitrary compliance. In addition, direct force control of the BWS system is necessary to avoid creating an extra constraint. Both compliance control and constraint release depend on the force controllability of the robot. Therefore, the generating methods of $\tau_{EXO}$ and $F_{BWS}$ are described in detail in the next section.

## 3 Actuation design

As the core element of ChMER, proper actuation is essential to improve the compliance and transparency of the robot. However, it is difficult for the robot to achieve complete transparency in practice, requiring a multifactorial trade-off. Therefore, this section discusses the actuation design analysis of the exoskeleton and the BWS system.

### 3.1 Actuation of the exoskeleton joint

**FIGURE 4:**
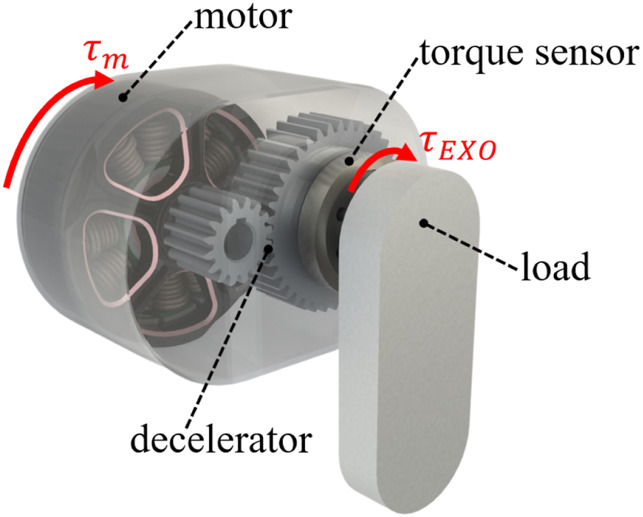
A typical exoskeleton joint.

A typical exoskeleton joint is shown schematically in Figure 4. The expression for its output torque $\tau_{EXO}$ is as follows:

$$\tau_{EXO}=\tau_{m}\cdot n-I_{total}\cdot \ddot{\theta }-f_{m}\left(\dot{\theta }\right)$$ (4)

$$\tau_{m}=i_{m}\cdot K_{t},I_{total}=I_{m}\cdot n^{2}+\Sigma I_{j}\cdot {n_{j}}^{2}$$ (5)

where n is the transmission ratio, $\tau_{m}$ is the torque generated by the motor, $i_{m}$ is the motor current, $K_{t}$ is the torque constant, $I_{m}\cdot n^{2}$ is the reflective/inertia of the motor rotor, $I_{j}$ and $n_{j}$ are the inertia and ratio of each gear so that $\Sigma I_{j}\cdot {n_{j}}^{2}$ is the combined reflective inertia of the gearbox, and $f_{m}\left(\dot{\theta }\right)$ is the friction. As for a TSA with large mechanical impedance, $\tau_{EXO}$ is usually measured by the torque sensor mounted between the exoskeleton joint and the load (such as Figure 4). In contrast to the external sensor-based scheme, assuming that $I_{total}\cdot \ddot{\theta }$ and $f_{m}\left(\dot{\theta }\right)$ are identified. Then, $\tau_{EXO}$ can be calculated by measuring $i_{m}$ with an internal current sensor and substituting it into Equation 4 without the need for a torque sensor. This method is also known as the proprioceptive sensing (Seok et al., 2012). Equations 4, 5 also show that the accuracy of proprioceptive sensing, as well as the transparency, depends on a variety of factors, such as the transmission ratio, reflective inertia, and friction.

In terms of improving the transparency of the exoskeleton joint, the transmission ratio n needs to be minimized because the larger it is, the less accurate the identification of $I_{total}$ and $f_{m}\left(\dot{\theta }\right)$ . However, if n is too small, the exoskeleton joint cannot meet the output torque requirement of the robot. Improving $\tau_{m}$ can also increase $\tau_{EXO}$ , but this requires expanding the air gap radius of the motor, which adds to the size and weight of the exoskeleton joint. QDD actuators choose a compromise between output capability, transparency, and size, which use a low gear ratio ( n≤10 ) to increase the output torque as well as maintain a low mechanical impedance for dynamic legged motion with accurate proprioception. QDD actuators were initially used in legged robots (Katz et al., 2019) and subsequently introduced into wearable devices. However, the QDD actuators developed for exoskeletons in current research have too little output torque (Zhu et al., 2019) or are too large (Yu et al., 2020) to meet the requirements in Table 2. Considering the advantages of high compactness, proprioception, and low cost of the QDD paradigm (Katz et al., 2019), the exoskeleton joint of ChMER adopts similar actuation, with the main difference of adding an extra stage of planetary gear reducer to guarantee the output torque.

Due to the small acceleration $\ddot{\theta }$ of rehabilitation, the negative influence of the reflective inertia $I_{total}\cdot \ddot{\theta }$ is diminished. The friction $f_{m}\left(\dot{\theta }\right)$ can also be partially compensated by identification. Therefore, although the secondary decelerator reduces some transparency, we still use the proprioception to estimate and control $\tau_{EXO}$ . It is first converted to $i_{m}$ by the following equation:

$$i_{m}=\frac{\tau_{EXO}+f_{m}\left(\dot{\theta }\right)}{K_{t}\cdot n}$$ (6)

Then the closed-loop control of $i_{m}$ is implemented. It can be seen that the control method of $\tau_{EXO}$ is actually open-loop. Since the current control loop of $i_{m}$ usually has high performance, the control accuracy of $\tau_{EXO}$ mainly depends on the precision of proprioception, namely, the transformation of Equation 6, which is verified in the experimental section later.

### 3.2 Design of the exoskeleton joint

**FIGURE 5:**
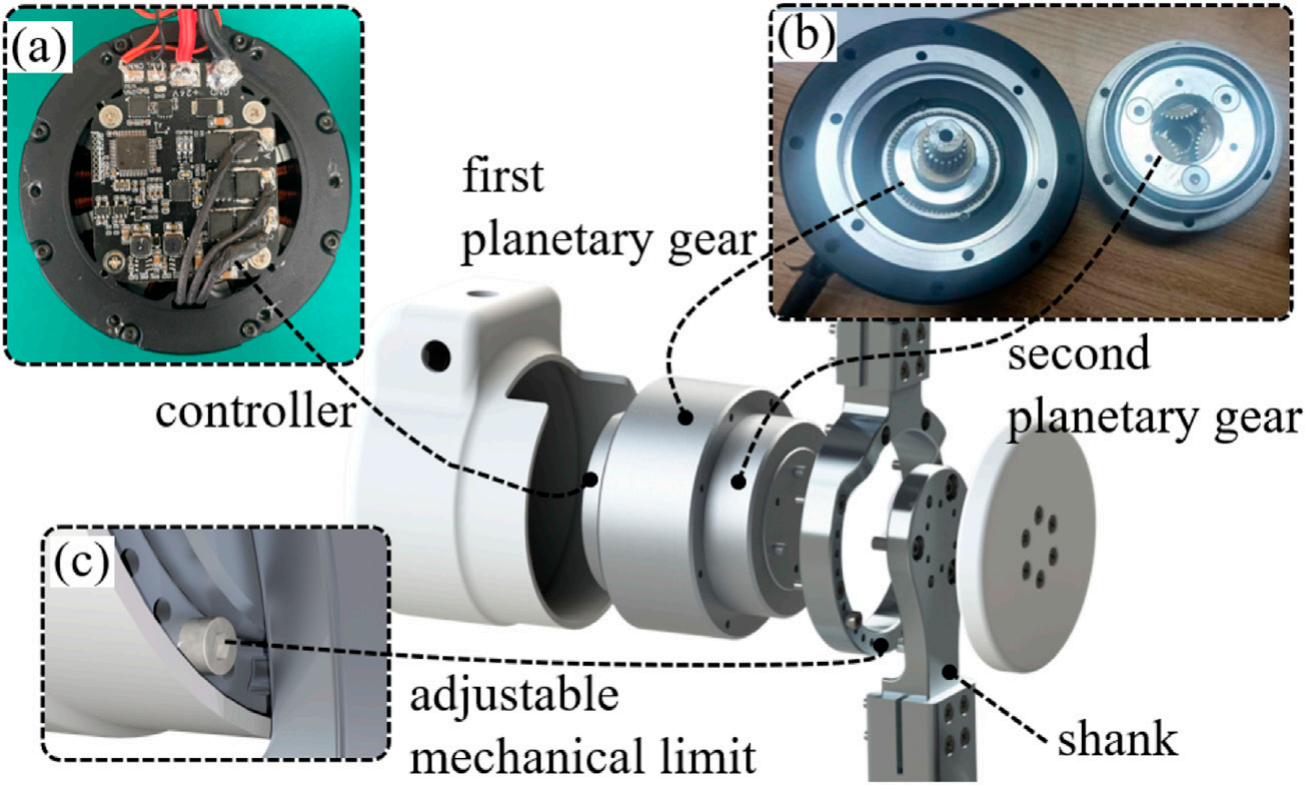
Overall design of the exoskeleton joint. **(A)** Controller. **(B)** Secondary planetary reducer. **(C)** Adjustable mechanical limit for different ranges of joint motion of children with CP.

The design requirement of the exoskeleton joint is to have enough output torque without excessively increasing the mechanical impedance to maintain the backdrivability and transparency. Therefore, based on the analysis in Section 3.1, we adopt the actuator (customized from Haitai Electromechanical, China) that uses a secondary planetary reducer (Figure 5B) with a total transmission ratio of 36:1. The nominal torque of the actuator is 18 Nm, the peak torque is 42 Nm, and the backdriving torque is tested to be within 1 Nm, with the mass of 580 g and the size of ϕ76 mm×52 mm . The overall design of the exoskeleton joint is shown in Figure 5, which consists of an actuator, a controller, shanks, mechanical limits, and other parts. Since children with CP have different ranges of joint motion, the mechanical limit (Figure 5C) can be adjusted by locking the limit screw into different threaded holes. The custom controller (Figure 5A) is mounted behind the motor and detects the angle through a magnetic encoder. Due to the exoskeleton operating primarily at low speeds during rehabilitation, an 18-bit resolution magnetic encoder (MT6825, MagnTek) is used to improve speed detection accuracy. Given that current detection accuracy directly determines the performance of the $i_{m}$ closed-loop control, high-precision current detection amplifier chips (INA181, Texas Instruments) are used for current sampling. Finally, due to the 36:1 transmission ratio, a wrong zero position may be recognized after re-powering up. Therefore, an inertial sensor (MPU6050, InvenSense) is used to detect the initial inclination of the exoskeleton to find the correct zero position.

### 3.3 Actuation of the BWS system

**FIGURE 6:**
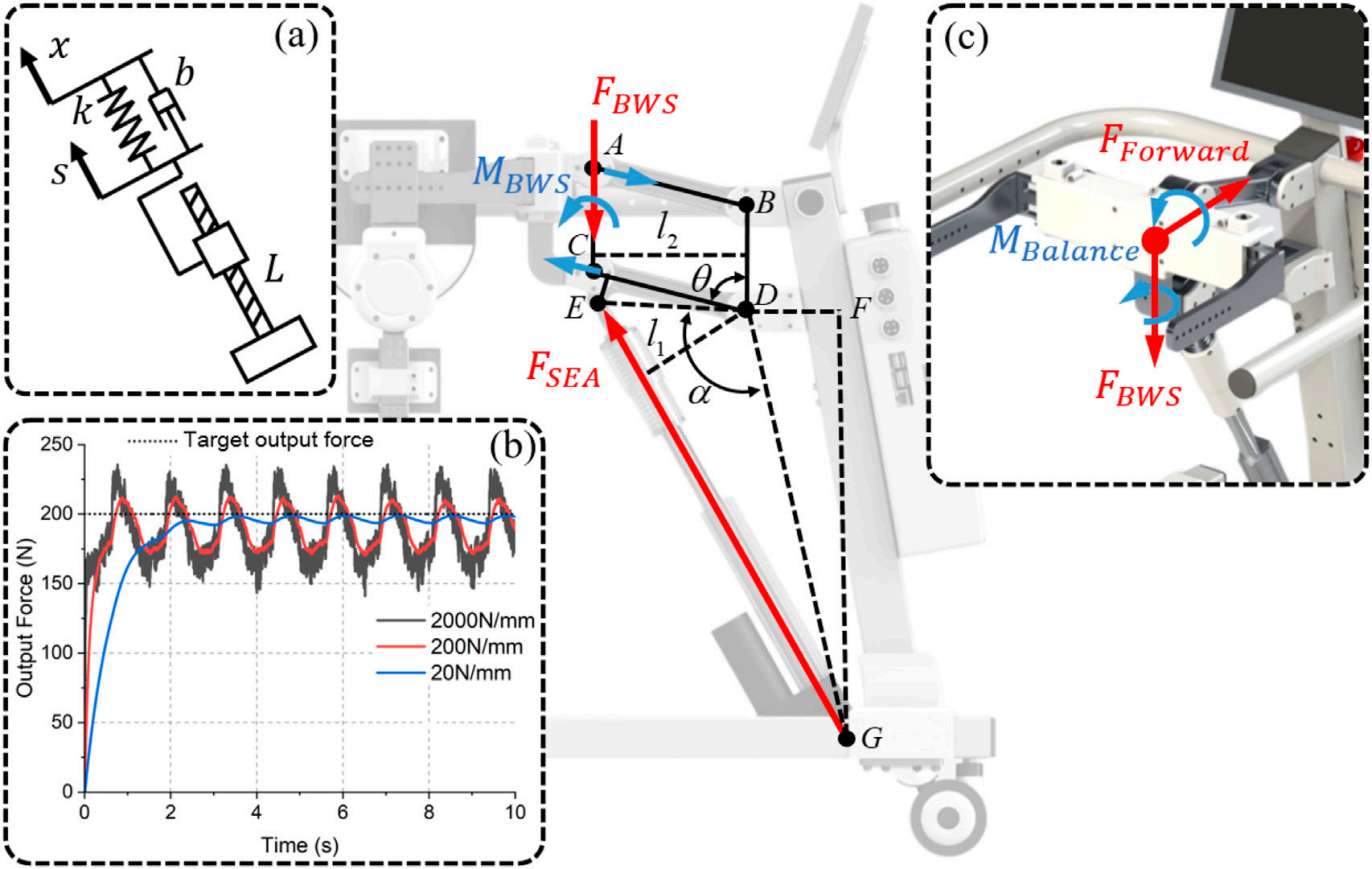
Analysis of the BWS system. **(A)** Diagram of the connection between the linear actuator and the load. **(B)** The simulation results of tracking 200 N for different k . **(C)** Disturbance moments and forces to the vertical support force $F_{BWS}$ .

The expression for the output force F of a linear actuator is as follows:

$$F=K_{t}\cdot i_{m}\cdot \left(2\pi /P\right)-I_{m}\cdot \left(2\pi /P\right)\ddot{s}-f_{m}\left(\dot{s}\right)$$ (7)

$$U-U_{b}=L\frac{di_{m}}{dt}+R\cdot i_{m}$$ (8)

$$U_{b}=K_{b}\cdot s\cdot \left(2\pi /P\right)$$ (9)

where P is the lead of the screw inside the linear actuator, s is the displacement, $K_{b}$ is the back-electromagnetic constant, U is the input voltage, $U_{b}$ is the back-electromagnetic voltage, L is the inductance, and R is the resistance of the motor. Usually, P is only a few millimeters to ensure sufficient F , so the generalized transmission ratio 2π/P is large, making the linear actuator’s mechanical impedance too high to be backdriven. Obviously, it cannot adopt open-loop force control. As shown in Figure 6A, assuming that the connection stiffness between the linear actuator and the load is k and the connection damping is b , the output force F can be expressed again as follow:

$$F=k\left(s-x\right)+b\left(\dot{s}-\dot{x}\right)$$ (10)

where x is the position determined by the fluctuation of the system’s CoMH. Combining Equations 7–10, assuming that x oscillates with a sinusoidal undulation and using a PID controller to adjust the input voltage U to track the desired F , the simulation results for different k are shown in Figure 6B. It can be seen that the smaller the stiffness k , the easier it is to control F , but it also reduces the rapidity of the system. Assuming a large k , a small s will result in a tremendous change of the F . However, the dynamic performance of the linear actuator is usually too poor to quickly adjust the s due to the large reflective inertia $I_{m}\cdot \left(2\pi /P\right)$ and friction $f_{m}\left(\dot{s}\right)$ . Thus, it is impossible to directly control F with a large k , which would otherwise lead to system instability (like 2000 N/mm in Figure 6B). Therefore, an SEA is finally used as the actuator, which reduces the connection stiffness by adding a spring between the linear actuator and the load, thus allowing direct feedback control of the F .

Although the control object $F_{BWS}$ can be measured directly by arranging the sensor at the point in Figure 6C, it will be disturbed by other moments and forces, such as $M_{Balance}$ and $F_{Forward}$ . Placing the sensor there will also cause difficulties in structural integration. As shown in Figure 6, the balance moment provided to the user can be counteracted by the four-bar structure without disturbing the output force $F_{SEA}$ of the SEA. In addition, the gravity and inertia of the four - bar structure can be neglected due to the small mass of the four - bar structure. Therefore, $F_{SEA}$ is only affected by $F_{BWS}$ , so we choose to measure $F_{SEA}$ and convert it to $F_{BWS}$ using the following equation:

$$F_{BWS}l_{2}=F_{SEA}l_{1}$$ (11)

The angle θ of the four-bar structure can be obtained by measurement, and the dimensional parameters of the bars are all known, so $l_{2}$ can be calculated by:

$$l_{2}=l_{CD}\sin\theta$$ (12)

∠CDE and ∠FDG can be obtained from the CAD model so that the angle α can be calculated by:

$$\alpha =\frac{3}{2}\pi -\theta -∠CDE-∠FDG$$ (13)

$l_{ED}$ and $l_{DG}$ can be obtained from the CAD model as well, so $l_{EG}$ can be calculated by:

$$l_{EG}=\sqrt{{l_{ED}}^{2}+{l_{DG}}^{2}-\cos\alpha \cdot 2l_{ED}l_{DG}}$$ (14)

Finally, by combining Equations 13–15, the length of $l_{1}$ can be calculated as Equation 16 so that $F_{BWS}$ can be finally converted to $F_{SEA}$ according to Equation 11.

$$l_{EG}l_{1}=l_{ED}l_{DG}\sin\alpha$$ (15)

$$l_{1}=\frac{l_{ED}l_{DG}\sin\alpha }{\sqrt{{l_{ED}}^{2}+{l_{DG}}^{2}-2\cos\alpha l_{ED}l_{DG}}}$$ (16)

In controlling $F_{BWS}$ , it is also necessary to first convert it to $F_{SEA}$ based on the above model and then implement the closed-loop control of $F_{SEA}$ . This further indicates the necessity of keeping the structure of the BWS system simple and efficient, as it improves the accuracy of the above modeling and, thus, the control accuracy of $F_{BWS}$ .

### 3.4 Design of the BWS system

**FIGURE 7:**
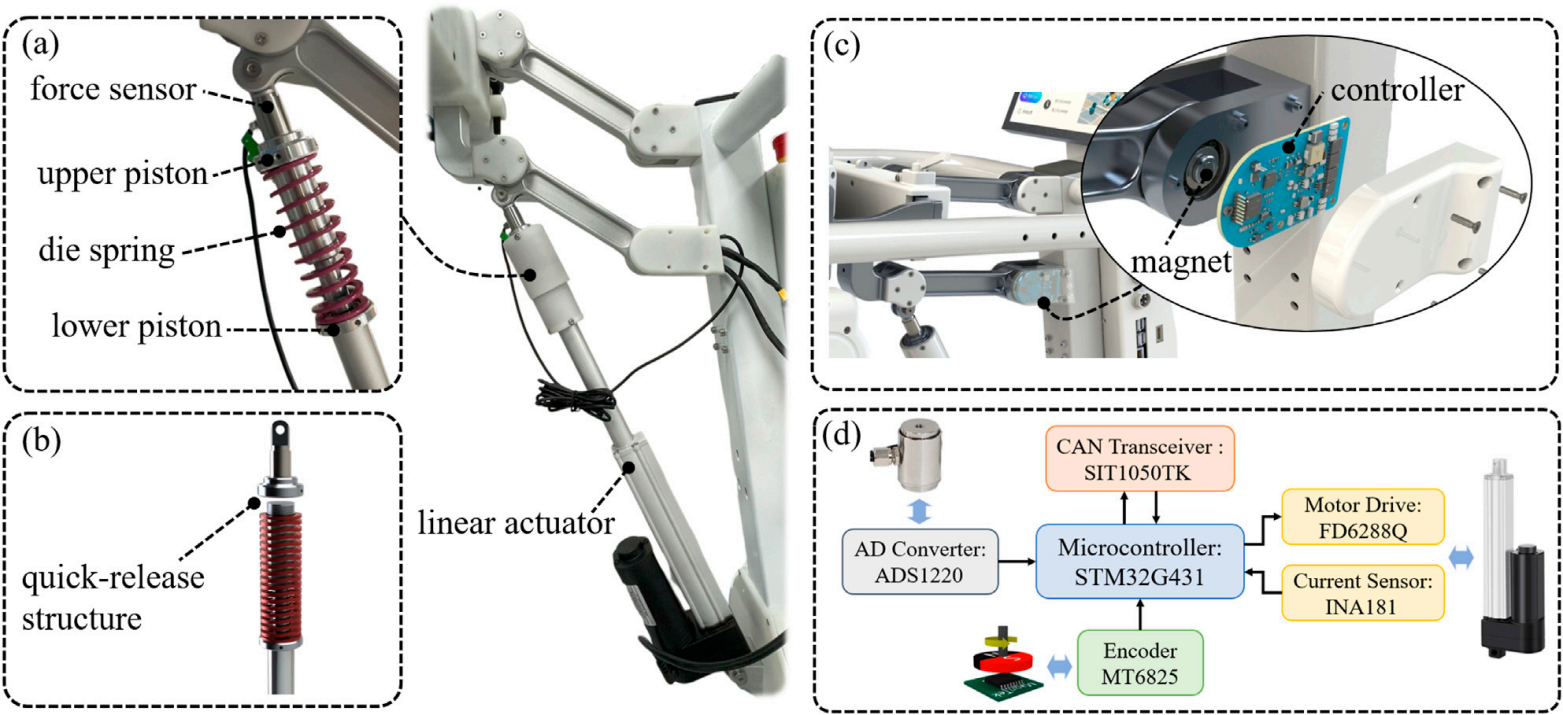
Overall design of the BWS system. **(A)** Design of the SEA; **(B)** Quick-release structure of the SEA for easy spring replacement. **(C)** Controller mounted on the rotary joint. **(D)** The hardware structure of the controller.

The design of the SEA is shown in Figure 7A, which includes a linear actuator (customized from Hoodland, China), a die spring, a piston device, and a force sensor (DYZ-102, DAYSENSOR). Usually, SEAs realize force sensing by measuring the compression of the elastic element. However, in order to improve compactness, a force sensor is used instead of the displacement sensor that requires a large installation volume. It can be seen from Equation 10 that the compliance and dynamic performance of the SEA are related to the stiffness of the elastic element. For children with severe motor injuries, the stiffness needs to be reduced to improve compliance and comfort. For children with partial motor abilities, the stiffness needs to be increased to accommodate relatively more dynamic movements. Therefore, a quick-release structure (Figure 7B) is adopted to conveniently replace the die springs that are available in a variety of stiffnesses with the same size specification to meet different children’s needs. In order to realize the highly mechatronic design of the BWS system, as shown in Figure 7C, the custom controller is mounted on the rotary joint of the four-bar structure. Therefore, the angle θ , which is used for the conversion of $F_{BWS}$ , can be detected by a magnetic encoder. The hardware structure of the controller is shown in Figure 7D, which integrates the functions of force sensor signal detection, angle detection, motor control, and controller area network (CAN) communication.

## 4 Control system

This section presents the hardware architecture of the control system and the control methods for passive and active rehabilitation. The exoskeleton and the BWS system are designed with transparency in mind. This design allows the transformation of $\tau_{EXO}$ and $F_{BWS}$ into closed-loop control of $i_{m}$ and $F_{SEA}$ , effectively simplifies the control. It also provides support for multiple active rehabilitation control strategies, as many studies have shown that the active participation of patients can help improve the rehabilitation effect (Liang et al., 2024; Tian et al., 2024). As one implementation of the strategies, the exoskeleton adopts an assisted-as-needed (AAN) strategy based on impedance control.

### 4.1 Hardware architecture

**FIGURE 8:**
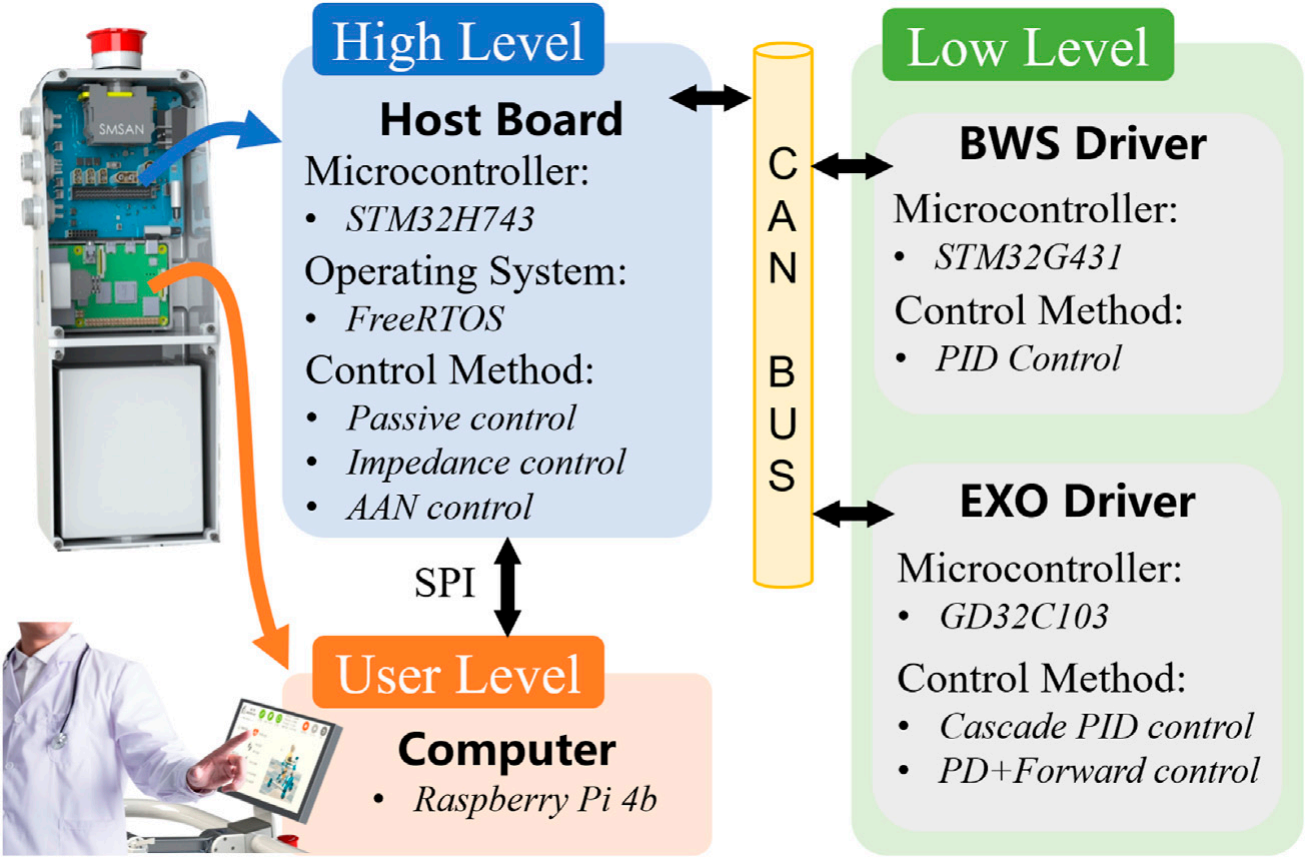
Hardware architecture of the control system.

A three-level control structure is adopted to ensure security during rehabilitation, as shown in Figure 8, including the low-level, high-level, and user-level. At the user level, a Raspberry Pi 4b is responsible for human-computer interaction and data storage. The user level communicates with the high-level host board via the serial peripheral interface (SPI) bus to send basic commands such as start-stop and to obtain robot status information. All real-time motion controllers run on the host board that uses a high-performance microcontroller (STM32H743, STMicroelectronics) and FreeRTOS as the real-time operating system. In this way, the isolation between the user level and the high level is achieved so that the motion controllers, which are directly related to safety, are not affected by the user-level software. The low level contains the drivers for the exoskeleton joints and the BWS system, which receive and execute motion control commands from the host board and return status information via the CAN bus.

### 4.2 Control architecture

**FIGURE 9:**
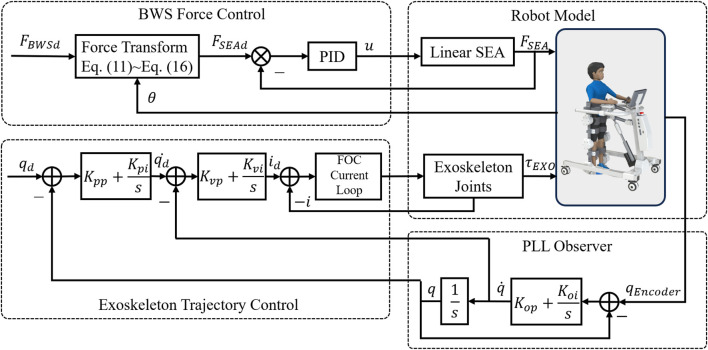
Control block diagram for passive rehabilitation.

The control block diagram for passive rehabilitation is shown in Figure 9. The BWS system tracks a predefined support force $F_{BWSd}$ , which is first converted to the desired $F_{SEAd}$ according to Equations 11–16 and then controlled by a PID controller. Due to the random detection errors of the joint encoder and the low operating speed of the exoskeleton, which result in a high level of noise in the velocity detection, a phase-locked loop (PLL) observer is used to filter and estimate the velocity of the exoskeleton joints. The exoskeleton performs the trajectory tracking control, using a fitted Fourier series of healthy children gait data (Gage and Novacheck, 2001) as the reference trajectory. A cascaded PID controller consisting of the position, velocity, and current loops is used to improve tracking accuracy. The desired current $i_{d}$ input to the current loop can be calculated by Equation 17.

$$i_{d}=\left[\left(q_{d}-q\right)\left(K_{pp}+\frac{K_{pi}}{s}\right)-\dot{q}\right]\left(K_{vp}+\frac{K_{vi}}{s}\right)$$ (17)

**FIGURE 10:**
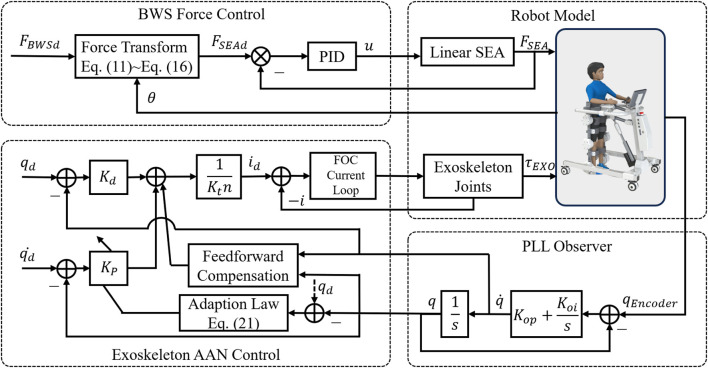
Control block diagram for active rehabilitation.

The control block diagram for active rehabilitation is shown in Figure 10. The BWS system still uses the above control method, while the exoskeleton adopts the proprioception-based impedance control. Let the assistive torque $\tau_{EXO}$ of the joints satisfy the impedance control expression and compensate for the gravitational moments of the exoskeleton. The desired torque of the joints is obtained as follows:

$$\tau_{EXOd}=K_{p}\left(q_{d}-q\right)+K_{d}\left(\dot{q_{d}}-\dot{q}\right)+G_{robot}\left(q\right)$$ (18)

where $K_{p},{\;K}_{d}$ are the virtual spring stiffness and virtual damping, q is the joint angle, $G_{robot}\left(q\right)$ is the gravity compensation matrix of the exoskeleton as shown in the following equation:

$$G_{robot}\left(q\right)=\left[\begin{array}{l} m_{1}gd_{1}S_{q1}+m_{2}g\left(l_{1}S_{q1}+d_{2}S_{q1+q2}\right)+m_{3}g\left(l_{1}S_{q1}+l_{2}S_{q1+q2}+d_{3}S_{q1+q2+q3}\right)\\ m_{2}gd_{2}S_{q1+q2}+m_{3}g\left(l_{2}S_{q1+q2}+d_{3}S_{q1+q2+q3}\right)\\ m_{3}gd_{3}S_{q1+q2+q3} \end{array}\right]$$ (19)

where m,d,l are the mass, center of mass length, and link length parameters, $S_{q1+q2}$ represents $\sin\;\left(q_{1}+q_{2}\right)$ . Thus, according to Equations 6, 18, 19, the desired current of the motor can be calculated by Equation 20.

$$i_{d}=\frac{\tau_{EXOd}+f\left(\dot{q}\right)}{K_{t}n}$$ (20)

where $K_{t}$ is the torque constant of the motor and n is the transmission ratio.

Based on the impedance control, an AAN strategy (Maggioni et al., 2018) is implemented to automatically adjust the impedance parameters according to the joint tracking errors that relate to the patient’s training performance. The virtual spring stiffness is updated by the tracking errors with the adaptation law as follows.

$$K_{p\_new}=\gamma \cdot K_{p\_last}+g\cdot \left|q_{d}-q\right|$$ (21)

where γ is the forgetting factor ( γ<1 ), and g is the error gain. Thus, for larger joint tracking errors, which represent that the patient has poor motor ability, the virtual spring stiffness $K_{p\_new}$ is raised to increase the assistive torque, while for smaller tracking errors, $K_{p\_new}$ is lowered to decrease the assistive torque due to the forgetting factor.

## 5 Experimental results and discussion

The test performance of the robot can be seen in the Supplementary Video S1.

### 5.1 Exoskeleton joint evaluation

**FIGURE 11:**
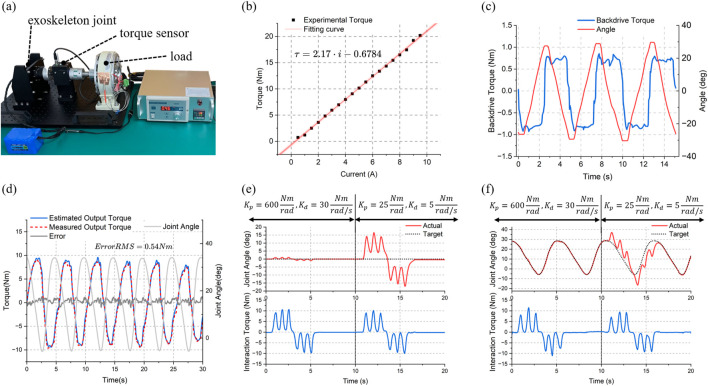
Test results of the exoskeleton joint. **(A)** Test platform. **(B)** Current versus torque curve. **(C)** Backdrivability test (within 1 Nm backdriving torque). **(D)** Proprioceptive torque sensing accuracy test (0.54 Nm RMS estimated error). Impedance control test of the joint under **(E)** static condition and **(F)** dynamic condition.

In order to evaluate the negative effect of the secondary planetary reducer on the transparency of the exoskeleton joint, a test platform shown in Figure 11A is constructed using a torque sensor (HLT-171, Hualiteng Technology) with a measurement accuracy of 0.3% F.S. to measure the actual output torque. First, the torque constant of the motor is calibrated, and the result is shown in Figure 11B, which shows that the output torque has a high linear correlation with the current. The backdrive torque of the exoskeleton joint, which is in the unpowered mode during the measurement, is shown in Figure 11C. It is within ±1Nm when the exoskeleton joint is manually rotated back and forth at the output side with a cycle of about 5 s. Finally, the proprioceptive sensing accuracy is tested under dynamic conditions. The interaction torque is applied to the output side by the human hand while the exoskeleton joint is rotated with the hip gait curve. The estimated interaction torque (blue solid line) and the measured torque sensor value (red dashed line) are shown in Figure 11D, where the estimated value is calculated by substituting the actual motor current into Equation 6. The root mean square (RMS) error is 0.54 Nm, which is about 5.4% of the peak amplitude.

The above results indicate that even with the additional impedance from the secondary planetary gear, the exoskeleton joint maintains high mechanical compliance. After friction compensation under walking - speed conditions, it also demonstrates high proprioceptive sensing accuracy. This provides a basis for impedance control. The test results of a single exoskeleton joint under static and dynamic conditions are presented in Figure 11E and (f) respectively. The first 10 s were set with larger virtual spring and damping parameters ( $K_{p}=600Nm/rad,{\;K}_{d}=30Nm\cdot s/rad$ ), while the parameters in the second 10 s were smaller ( $K_{p}=25Nm/rad,{\;K}_{d}=5Nm\cdot s/rad$ ). It can be observed that for nearly the same interaction torque, the exoskeleton joint exhibits significantly different impedance characteristics. This verifies the feasibility of proprioception - based compliance control for our exoskeleton.

### 5.2 Active BWS system evaluation

**FIGURE 12:**
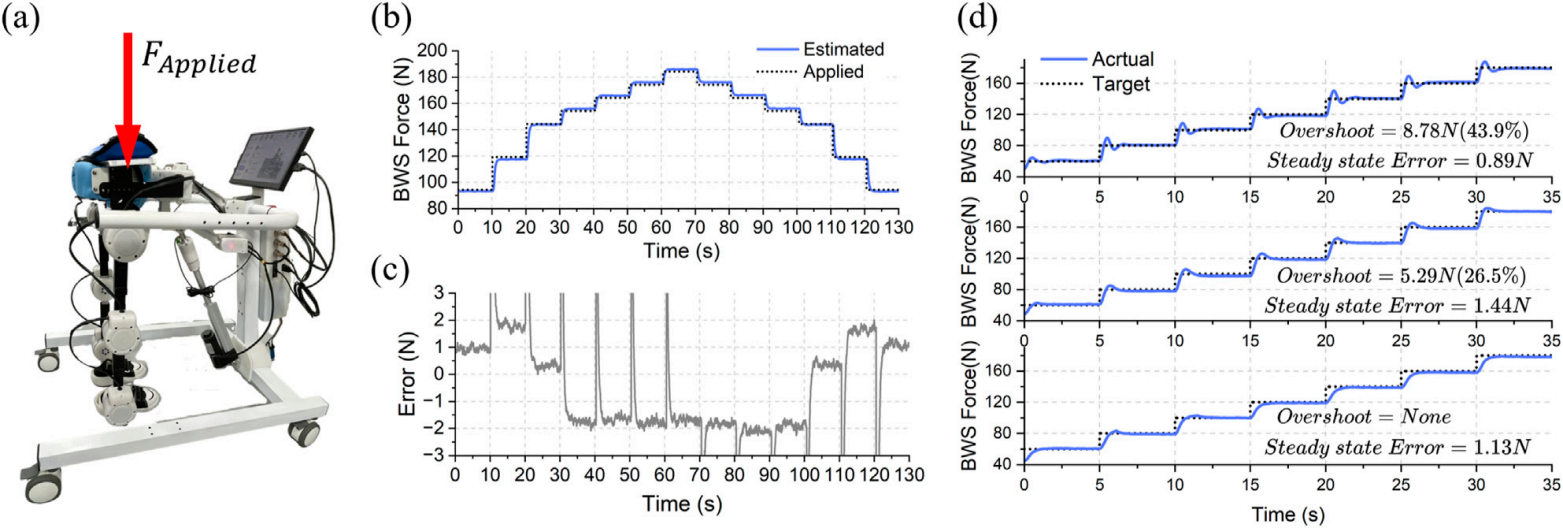
Test results of the BWS system. **(A)** Method of testing the accuracy of the conversion model by stacking weights as the reference force. **(B)** The estimated support force and actual force applied. **(C)** The error between estimated and applied force (within about ±2N). **(D)** The step response performance of the BWS system.

The accuracy of the conversion model of $F_{SEA}$ and $F_{BWS}$ proposed in Section 3.3 was first tested. The test method is depicted in Figure 12A. A series of standard mass weights were sequentially stacked to create an applied force $F_{Applied}$ of known magnitude. This $F_{Applied}$ was then used as the reference force (shown as the black dashed line in Figure 12B). The blue solid line in Figure 12B shows the estimated $F_{BWS}$ after conversion from $F_{SEA}$ . The difference between $F_{Applied}$ and $F_{BWS}$ , as shown in Figure 12C, reveals that the conversion error after stabilization is within about ±2N, with the spikes appearing due to the gradual stabilization of $F_{BWS}$ by the spring of the SEA after the weight is dropped momentarily. The step response performance of the BWS system was then tested under several sets of PID parameters. The desired $F_{BWS}$ was sequentially increased with an amplitude of 20 N at 5 s intervals. As shown in Figure 12D, the overshoots decrease sequentially from the top panel to the bottom panel (approximately 43.9%, 26.5%, and 0%), and the steady-state errors are all within 2 N (about 0.89, 1.44, and 1.13 N). These results demonstrate that the proposed BWS system exhibits low conversion and steady - state errors for $F_{BWS}$ . This provides a foundation for controlling $F_{BWS}$ during the dynamic walking process of ChMER, the performance of which is shown in the next section.

### 5.3 Experimental results of the passive rehabilitation control framework

**FIGURE 13:**
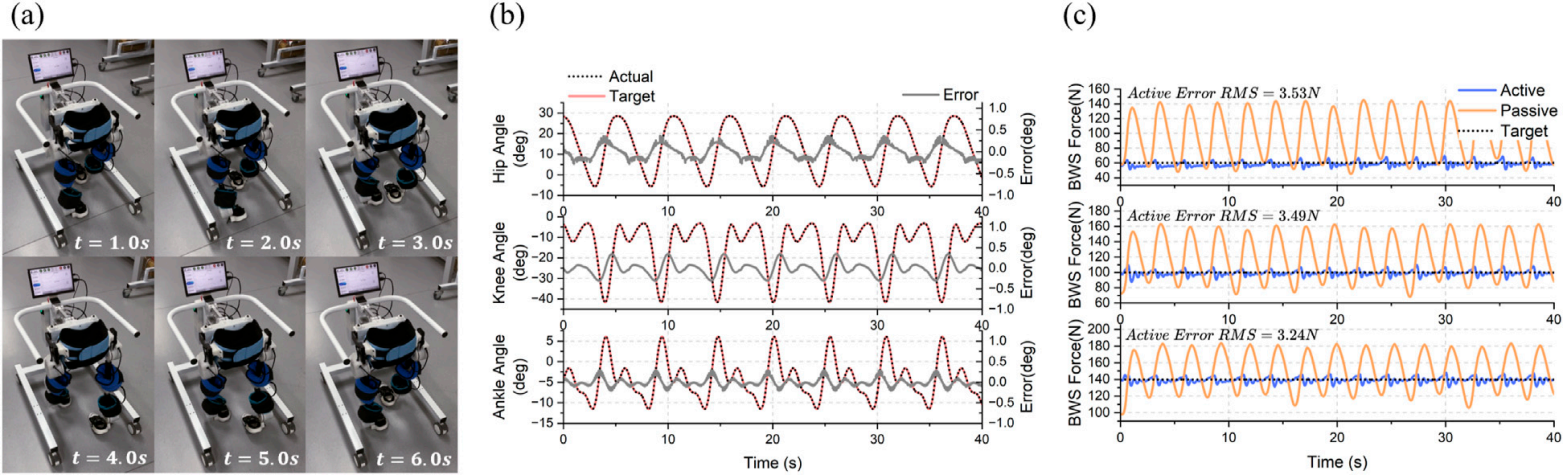
Experimental results of passive rehabilitation control framework. **(A)** Independent ground walking test of ChMER. **(B)** Trajectory tracking results (within ±0.5 degrees of error). **(C)** The active and passive support force during dynamic walking.

The performance of ChMER in passive rehabilitation is demonstrated in this section. Since the robot is still in the prototype validation phase, for safety reasons, sandbags were used to simulate the children with a load of approximately 3 kg on the thighs and 2.5 kg on the calves on each side. As described in Section 2.2, ChMER is designed to walk stably on its own. This ability is verified by the independent ground - walking test shown in Figure 13A. The trajectory tracking effect of the cascade PID controller is shown in Figure 13B, where the maximum tracking errors of each exoskeleton joint are within ±0.5°. Finally, during the dynamic process of the robot walking on the ground, the performance of the BWS system was tested by tracking the desired $F_{BWS}$ of 60, 100, and 140 N. The RMS errors between the desired $F_{BWS}$ (shown by the black dashed line in Figure 13C) and the actual $F_{BWS}$ (shown by the blue solid line in Figure 13C) are approximately 3.53, 3.49, and 3.24 N, respectively. The orange solid line in Figure 13C is the $F_{BWS}$ relying only on the passive flexibility of the spring when the SEA is in the unpowered mode. The variation of $F_{BWS}$ for the active support is significantly smaller than that for the passive support (reduced by about 80%). The above results indicate that the BWS system has high performance capabilities, meeting the requirements for constant weight unloading during gait rehabilitation. Additionally, the comparison experiment between active and passive support validates the design in Section 2.2, demonstrating that the BWS system can automatically adapt to the changing Center of Mass of the Human (CoMH). This reduces support force fluctuations and improves compliance, highlighting the advantage of the active BWS system.

### 5.4 Experimental results of the active rehabilitation control framework

**FIGURE 14:**
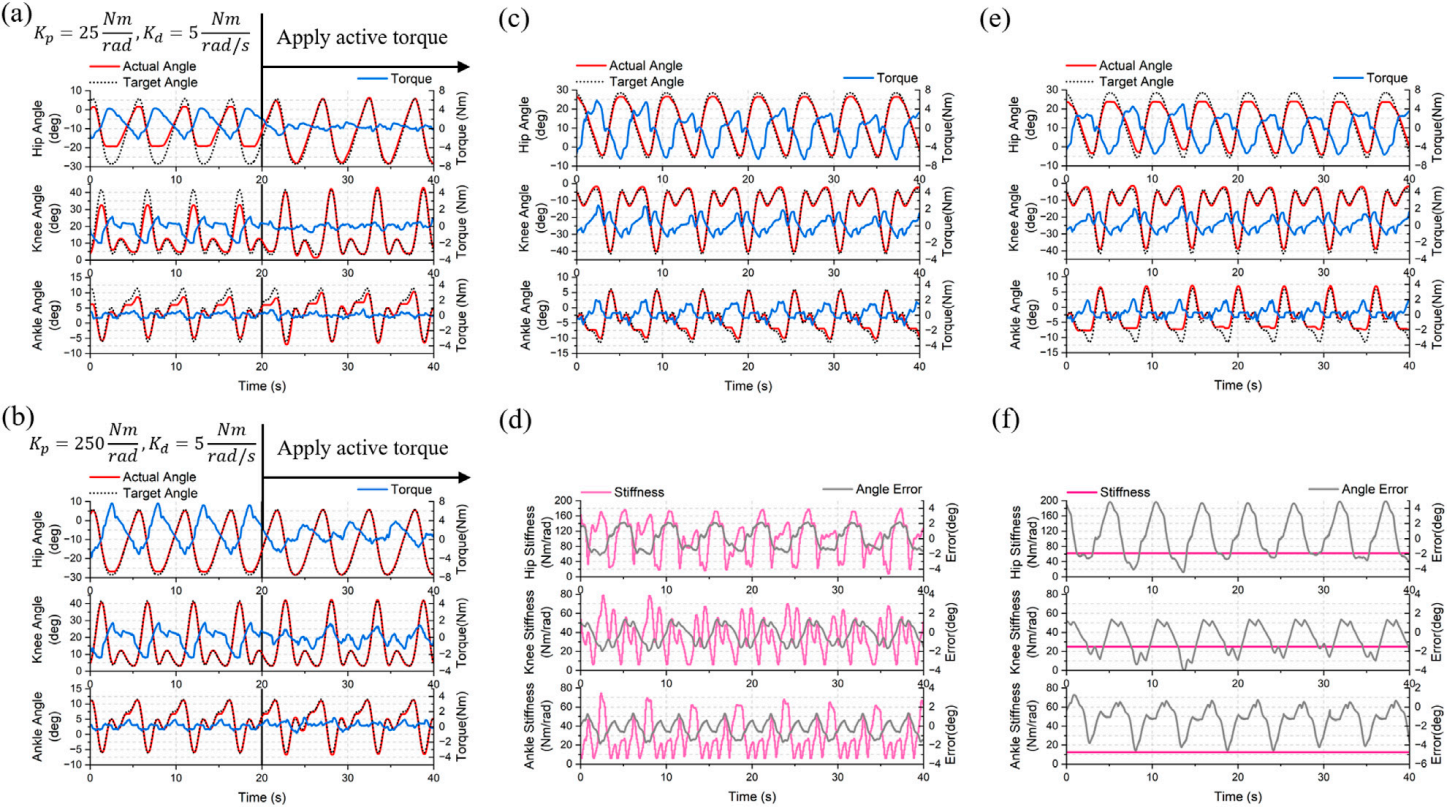
Experimental results of the active rehabilitation control framework. Impedance control with constant parameters of **(A)** $K_{p}=25\frac{Nm}{rad},K_{d}=5\frac{Nm}{rad/s}$ and **(B)** $K_{p}=250\frac{Nm}{rad},K_{d}=5\frac{Nm}{rad/s}$ . **(C)** The exoskeleton joint angle and assistive torque of AAN control. **(D)** The change in $K_{p}$ with trajectory tracking errors. Figures **(E)** and **(F)** are the contrast with constant $K_{p}$ .

The performance of ChMER in active rehabilitation under different constant impedance parameters is shown in Figures 14A, B. Due to the gravity of the sandbags used as the load, the exoskeleton deviated from the desired trajectory in the first 20 s. In the second 20 s, by using the hand to simulate the active torque of the user, the exoskeleton returned to the desired trajectory, and the assistive torque provided by the exoskeleton in Figure 14A was less than that in Figure 14B due to the smaller virtual spring stiffness $K_{p}$ . Finally, we tested the effect of the AAN control strategy based on Equation 21. The exoskeleton joint angle and assistive torque are shown in Figure 14C, and the change of $K_{p}$ with trajectory tracking errors is shown in Figure 14D, while the results without activating the AAN strategy are shown in Figures 14E, F. It can be seen that the AAN strategy adaptively increases the $K_{p}$ for large tracking errors, thus increasing the assistive torque and decreasing the tracking errors.

These results show that ChMER is capable of adjusting the assistive torque on demand. Although this AAN strategy simply modifies the impedance control parameters, its successful implementation also demonstrates that the proposed robot has the potential for further applications of intelligent AAN control algorithms, such as using Gaussian radial basis functions (RBFs) to identify the patient’s residual motor ability and adaptively modify the assistive torque (Luo et al., 2019; Wang et al., 2022).

## 6 Conclusion

In this work, we developed a novel mobile exoskeleton rehabilitation robot (ChMER) with an active BWS walker for young children (3- ∼ 6 years-old) with CP. ChMER has high compliance while maintaining a compact structure to accommodate the small limbs of young children. We proposed a compact kinematic chain that integrates an exoskeleton, an active BWS system, and a walker. With the analysis and appropriate actuation setting of the kinematic chain, ChMER is able to walk stably on its own to ensure the safety of passive rehabilitation. It can also adapt to the varying CoMH thanks to the active BWS system, which significantly reduces the fluctuation (about 80%) of the weight support force compared to the passive BWS, thus improving the compliance. Based on the intrinsically compliant actuation, ChMER also supports compliant force control in active rehabilitation. The exoskeleton joint, inspired by the QDD paradigm, maintains high mechanical compliance (1 Nm backdrive torque) and uses a secondary planetary reducer (ratio = 36:1) to ensure high output torque (18 Nm nominal torque). It also has a high proprioceptive torque sensing accuracy of 0.54 Nm RMS error (5.4% of the peak amplitude) under walking speed conditions to realize assistive torque control, which can replace the torque sensor and help reduce the cost and complexity of the robot. The BWS system uses an SEA to accurately generate the support force with 3.53/3.49/3.24 N RMS tracking errors for desired support forces of 60/100/140 N during dynamic walking. Finally, an AAN control strategy based on impedance control is applied as an implementation of active rehabilitation control. ChMER exhibits the desired compliance, demonstrating its potential for further applications of intelligent AAN control algorithms.

The limitation of this work is that there is no performance study with real users, as it focuses on design validation and performance testing of the proposed rehabilitation robot. In future work, we will further improve the structure of the robot and investigate the performance of real users, such as kinetics and electromyography, and validate the effectiveness of the robotic rehabilitation therapy on children with CP. The proposed rehabilitation robot can potentially provide a more effective and convenient rehabilitation solution for children with CP.

## Data Availability

The original contributions presented in the study are included in the article/Supplementary Material, further inquiries can be directed to the corresponding author.
